# Human Umbilical Cord Mesenchymal Stem Cell Differentiation Into Odontoblast-Like Cells and Endothelial Cells: A Potential Cell Source for Dental Pulp Tissue Engineering

**DOI:** 10.3389/fphys.2020.00593

**Published:** 2020-06-23

**Authors:** Shuang Zhang, Weiwei Zhang, Yanping Li, Liping Ren, Haotian Deng, Xiaowei Yin, Xu Gao, Shuang Pan, Yumei Niu

**Affiliations:** ^1^Department of Endodontics, The First Affiliated Hospital of Harbin Medical University, Harbin, China; ^2^Department of Endodontics, School of Stomatology, Harbin Medical University, Harbin, China; ^3^Department of Prosthodontics, The First Affiliated Hospital of Harbin Medical University, Harbin, China; ^4^Department of Biochemistry and Molecular Biology, Harbin Medical University, Harbin, China

**Keywords:** human umbilical cord mesenchymal stem cells, odontoblast-like cells, endothelial differentiation, angiogenesis, Hif-1 signaling pathway, dental pulp tissue engineering

## Abstract

**Objectives:**

Dental pulp regeneration is considered an ideal approach for treating dental pulp disease. Because pulp is composed of various cells, determining the proper seed cells is critical. We explored the potential of human umbilical cord mesenchymal stem cells (hUCMSCs) as seed cells for dental pulp regeneration.

**Methods:**

Liquid extract of human treated dentin matrix (LE-TDM) was acquired to culture hUCMSCs. Odontoblast-specific markers were detected by western blot, qRT-PCR, and immunofluorescence assays. Endothelial differentiation of hUCMSCs was examined according to VEGF induction by western blot, qRT-PCR, and Matrigel assays. hUCMSCs and VEGF-induced hUCMSCs (V-hUCMSCs) were also cocultured *in vivo* for the Matrigel plug assay and *in vitro* for RNA-sequencing (RNA-seq). Finally, encapsulated mono-cultured hUCMSCs or cocultured hUCMSCs and V-hUCMSCs in scaffolds were injected into the root segments and transplanted into immunodeficient mice for dental pulp regeneration.

**Results:**

Under LE-TDM induction, hUCMSCs expressed specific odontoblast markers (DSPP, DMP-1, DSP). Under VEGF induction, hUCMSCs expressed functional endothelial markers (CD31, eNOs, vWF). *In vivo*, the Matrigel plug assay indicated that cocultured hUCMSCs and V-hUCMSCs formed extensive vessel-like structures. RNA-seq results indicated that cocultured V-hUCMSCs exhibited high Hif-1 signaling pathway activity. Both the hUCMSCs mono-culture and coculture groups showed pulp-like tissue regeneration. The cocultured group showed more extracellular matrix and vascularization than the mono-cultured group *in vivo*.

**Conclusion:**

hUCMSCs can differentiate into odontoblast-like cells and functional endothelial cells. Cocultured hUCMSCs and V-hUCMSCs formed vessel-like structures and regenerated dental pulp-like tissue. Therefore, hUCMSCs can be used as an alternative seed cell source for angiogenesis and dental pulp regeneration.

## Introduction

The vitality of teeth is mainly maintained by dental pulp, which is responsible for the nerve supply, nutritional supply, and dentin production of teeth. However, the pulp is susceptible to insult by chemical, mechanical, thermal, or microbial irritants. Conventional root canal therapy replaces pulp tissue with artificial materials, without restoring the function of the dental pulp. The loss of dental pulp in young permanent teeth will attenuate root development and can even lead to tooth loss (Huang, 2008; Kim et al., 2010). Restoring the function of pulp tissue through tissue engineering technology can solve these problems (Huang, 2008, 2009; Xuan and Li, 2018). A major challenge in regenerating functional dental pulp is the reconstruction of a complex, highly organized histological tissue structure, which contains several types of cells in different regions and areas of vascularization (e.g., odontoblasts in the peripheral lining against the dentinal wall and the microvasculature in the central region). The key to the regeneration of dental pulp is identifying the most suitable seed cells.

In recent years, the application of dental stem cells in pulp regeneration has made remarkable achievements. However, there are several potential limitations to apply dental stem cells (the limited availability of dental stem cells). Therefore, a more suitable and practical kind of seed cell pulp regeneration is needed. Since mesenchymal stem cells (MSCs) were isolated from the human umbilical cord in 2003 (Romanov et al., 2003), significant concerns regarding the use of human umbilical cord mesenchymal stem cells (hUCMSCs) have been raised, but many advantages of these cells have been recognized. First, hUCMSCs have high proliferative capability and multilineage differentiation potential (Karahuseyinoglu et al., 2007). Second, their supply is not limited (Butler and Menitove, 2011; Gong et al., 2012). More importantly, compared to other sources of adult stem cells, there is a lower risk of viral contamination due to the protection provided by the placental barrier (Duya et al., 2013). In addition, studies have shown that hUCMSCs can differentiate into chondrocytes, osteoblasts, cardiomyocytes, and neurons, and hUCMSCs have been applied for cartilage regeneration, and bone tissue regeneration, and to treat kidney injury (Wang et al., 2016; Day et al., 2018; Wu et al., 2018; Zhang et al., 2018; Zheng et al., 2019). However, whether hUCMSCs can be applied in dental pulp regeneration remains unknown.

The microenvironment of stem cells is termed the “stem cell niche,” which maintains their functions and characteristics, and guides stem cell differentiation (Gong et al., 2012). Liquid extract of human treated dentin matrix (LE-TDM) contains many growth factors and complex soluble signaling molecules, which could provide an odontogenic microenvironment. In addition, accumulating evidence has confirmed that treated dentin matrix can promote the odontogenic differentiation of other stem cells, such as dental follicle cells (DFCs) (Li et al., 2011). These findings, together with the known advantages of hUCMSCs, encouraged us to investigate whether hUCMSCs could be induced to differentiate along the odontoblast lineage when exposed to LE-TDM, for subsequent use as seed cells for dental pulp regeneration.

Rapid and effective angiogenesis is important for successful connective tissue regeneration. In engineered constructs, the main role of the vasculature is to deliver nutrients and oxygen from host cells and tissues to the cells in scaffolds (Jabbarzadeh et al., 2008). Although endothelial cells (ECs) have a long lifespan and are commercially available, these cells are not often used for tissue regeneration due to associated safety concerns. Primary cells are more biologically relevant than immortalized ECs for tissue regeneration (Chong et al., 2016). The discovery of a cell type capable of differentiating into functional vascular EC would be beneficial for the advancement of tissue engineering. The present study was performed to investigate whether hUCMSCs have the capacity to differentiate into ECs, and whether they have angiogenic potential under conditions of vascular endothelial growth factor (VEGF) induction for the regeneration of vascularized dental pulp-like tissue.

We first evaluated whether hUCMSCs have the capacity to differentiate into odontoblast-like cells under the microenvironment induced by LE-TDM *in vitro*, to confirm their angiogenic potential *in vivo*. Finally, we co-transplanted hUCMSCs and VEGF-induced hUCMSCs (V-hUCMSCs; V) to regenerate dental pulp-like tissue. In summary, we explored a desirable alternative seed cell source for angiogenesis and dental pulp regeneration.

## Materials and Methods

### Cell Isolation, Culture, and Identification

The experimental protocols were approved by the Ethics Committee of Harbin Medical University. We collected human umbilical cords from Caesarean section patients during term deliveries in the obstetrical department of the Second Affiliated Hospital of Harbin Medical University. hUCMSCs were isolated from human umbilical cords as reported previously (De Bruyn et al., 2011; Li et al., 2013) and cultured in Dulbecco’s modified Eagle’s medium/F12 (DMEM/F12) with 10% (v/v) fetal bovine serum (FBS, Gibco, Grand Island, NY, United States) and 1% penicillin-streptomycin (regular medium) at 37°C in a humidified atmosphere with 5% CO_2_. For most of the experiments, hUCMSCs were used at p3–4. Adipogenic differentiation was induced by adipogenic medium (AM) containing DMEM/F12 with 10% FBS, 1% penicillin-streptomycin, 0.5 μM isobutyl-methylxanthine (IBMX, Sigma), 50 μM indomethacin (Sigma), 0.5 μM dexamethasone (Sigma), and 5 μg/ml insulin (Sigma) for 14 days. The medium was changed every 2 days. Then, Oil Red O solution (Sangon Biotech) was used to stain cells. Osteogenic differentiation of hUCMSCs was induced according to the manufacturer’s instructions in adapted medium (OM) (Cyagen Biosciences Inc., Guangzhou, China). After 14 days of induction, the cells were incubated in Alizarin Red for 5 min at room temperature to examine extracellular matrix calcification. Flow cytometric analysis was used to examine the expression levels of various antigen markers in hUCMSCs at the third passage of culture. CD90, CD73, CD105, CD34, and CD45 (Biolegend) staining assays were performed according to the manufacturer’s protocol.

### The Biological Effects of the Liquid Extract of Human Treated Dentin Matrix (LE-TDM) on hUCMSCs

#### Preparation of LE-TDM

The premolars and third molars were extracted from patients for clinical reasons at the First Affiliated Hospital of Harbin Medical University after obtaining informed consent. The method used to prepare LE-TDM was reported previously (Peng et al., 2011; Jiao et al., 2014). Periodontal tissues were completely scraped off with a curette and ground along the tooth profile to remove the outer cementum and part of the dentin. Predentin and dental pulp tissues were removed using a mechanical method. Finally, the dentin matrix was immersed in deionized water for ultrasonic cleaning for 20 min. Then, the dentin matrix was soaked in 17% ethylene diamine tetra-acetic acid (EDTA, Sigma) for 5 min, washed in deionized water for 10 min by an ultrasonic cleaner, exposed to 10% EDTA for 5 min, and washed in deionized water for 10 min with an ultrasonic cleaner. Finally, it was exposed to 5% EDTA for 10 min and then washed in deionized water for 10 min by an ultrasonic cleaner. The dentin matrix was stored in sterile PBS with 1% penicillin-streptomycin for 72 h and then washed in sterile deionized water for 10 min by an ultrasonic cleaner. The dentin matrix was ground into powder, and DMEM/F12 was added to the powder at a ratio of 20 g powder per 100 ml of DMEM/F12. The dentin matrix was then incubated at 37°C for 1 week. Finally, a 0.22 μm filter was used to filter the samples.

#### Cytotoxicity of LE-TDM

MTT assay was used to assess cytotoxicity. The hUCMSCs were seeded in a 96-well culture plate at a density of 2 × 10^3^ cells per well and incubated for 24 h to allow for initial attachment. Then, the cells were exposed to LE-TDM with 10% FBS and 1% penicillin-streptomycin (LE-TDM media). After culturing for 1, 2, 3, 4, or 5 days, 5 g/l MTT solution was added to each well, followed by incubation for 4 h. The medium was removed, and 150 μl of dimethyl sulfoxide (DMSO) was added to each well. After the crystals were dissolved, absorbance at 490 nm was measured using a spectrophotometer (Tecan). hUCMSCs cultured in regular media were used as controls. To analyze the relative growth rate (RGR), the level of cytotoxicity was divided into five stages as reported previously (Sakai et al., 2011). The relative growth rate was calculated according to the ISO 10993 protocol as%RGR = (absorbance of text group/absorbance of blank group) × 100%. The calculated RGRs are summarized in Supplementary Table S1.

#### Cell Migration Assay

##### Wound healing experiments

hUCMSCs were seeded into six-well plates (5 × 10^4^ cells per well) and incubated for 24 h to allow for initial attachment. Next, the tip of a pipette was used to scratch and scrape the monolayer of each plate, followed by the plate being washed twice with PBS to remove the debris. An LE-TDM medium or a regular medium was then added to the wells. Cell migration was inspected at 0 and 24 h by light microscopy (Olympus).

##### Transwell assay

Next, 2 × 10^4^ hUCMSCs resuspended in 100 μl of a serum-free medium were seeded into the upper chambers of transwell plates (BD Bioscience). An LE-TDM medium was added to the lower chamber as the test group. A regular medium was added to the control group. After incubation for 24 h, the cells beneath the membrane were stained with 0.5% crystal violet and counted.

#### Cell Adhesion Assay

Culture plates (3.5 cm in diameter) were coated with 1 ml of fibronectin (10 μg/ml) (Sigma) at 4°C overnight. Then, the culture plates were washed three times with PBS and blocked with 0.5% bovine serum albumin (BSA) in a medium at 37°C for 1 h. Cells (test group: cells were exposed to an LE-TDM medium for 7 days; control group: cells were cultured in a regular medium) were seeded on the culture plates and incubated for 30 min. The plates were rinsed with PBS to remove non-adherent cells. The attached cells were fixed with 4% paraformaldehyde, stained with crystal violet, and counted.

#### Real-Time PCR Analysis

After exposure to the LE-TDM medium for 7 days, the cells were harvested by a TRIzol reagent (Invitrogen). Reverse transcription was performed using reverse transcriptase (Invitrogen). Subsequently, the cDNA was subjected to real-time PCR on a 7500 Real-time PCR Detection System (AB Applied Biosystems). The primer sequences are listed in Supplementary Table S2.

#### Western Blot Analysis

After 7 days of induction by the LE-TDM medium, the total cell proteins were prepared by extraction and lysed with a RIPA buffer containing a protease inhibitor. To detect MAPK pathway-related proteins, protein was collected from hUCMSCs after treating with the LE-TDM medium for 60, 30, 15, and 0 min. Cells in the 0 min group were cultured in a regular medium for 60 min. Phosphatase inhibitor (Beyotime Institute of Biotechnology Jiangsu, China) was added to the protein solution according to the manufacturer’s instructions. The total protein was extracted at specific time points. Nuclear and cytoplasmic proteins were extracted using a Nuclear and Cytoplasmic Protein Extraction Kit (Beyotime Institute of Biotechnology Jiangsu, China) according to the manufacturer’s protocol. Protein extracts were separated by 10% SDS–PAGE gels, transferred to PVDF membranes, blocked in 5% skim milk with TBST for 2 h, and incubated with primary antibodies in a refrigerator at 4°C overnight. After incubation with secondary antibodies, signals were visualized by electrochemiluminescence (ECL).

Primary antibodies used were DSP (Santa Cruz Biotechnology, 1:500), CollagenI (Abcam, 1:500), DSPP (Santa Cruz Biotechnology, 1:500), DMP-1 (Santa Cruz Biotechnology, 1:500), BMP-2 (Abcam, 1:500), GAPDH (Proteintech, 1:5,000), JNK/p-JNK (Cell Signaling Technology, Beverly, MA, 1:500), p38/p-p38 (Cell Signaling Technology, Beverly, MA, 1:500), ERK/p-ERK (Cell Signaling Technology, Beverly, MA, 1:500), Ve-cadherin (Abcam, 1:500), CD31 (Abcam, 1:500), VEGFA (Abcam, 1:500), Hif-1α (Proteintech, 1:500), β-actin (Zhongshan Golden Bridge Bio-technology, China, 1:1,000), and Histone3 (Abcam, 1:500). Secondary antibodies used were anti-mouse IgG (Zhongshan Golden Bridge Bio-technology, China, 1:1,000) and anti-rabbit IgG (Zhongshan Golden Bridge Bio-technology, China, 1:1,000).

#### Immunofluorescent Staining

After 7 days of exposure to the LE-TDM medium, the cells were fixed with 4% polyoxymethylene for 20 min, permeabilized with 0.1% Triton X-100, and then incubated with DSPP primary antibody at 4°C overnight. The next day, the cells were washed three times with cold PBS and then incubated with florescent secondary antibody (along with DAPI). The cells were visualized by confocal microscope. hUCMSCs cultured in a regular medium were used as controls.

### VEGF-Induced hUCMSCs (V-hUCMSCs) *in vitro*

Endothelial differentiation *in vitro* was performed as previously reported, with some modifications (Chen et al., 2009). The hUCMSCs were seeded in 3.5 cm culture dishes (2 × 10^4^ cells/cm^2^) and then cultured in an endothelial differentiation medium [containing DMEM/F12, 50 ng/ml VEGF (Recombinant Human Vascular Endothelial Cell Growth Factor, PHC9394, Invitrogen), 2% FBS, and 1% penicillin-streptomycin] for 7 days. First, real-time PCR assay was used to evaluate gene expression. Then, western blot assay was used to detect protein expression. The primer sequences are listed in Supplementary Table S2. Finally, the formation of vessel-like structures by VEGF-induced hUCMSCs (V-hUCMSCs) on the basement membrane matrix Matrigel (BD Biosciences) was observed through an *in vitro* Matrigel angiogenesis assay. Briefly, V-hUCMSCs (1.5 × 10^4^ cells per well) were seeded in 96-well plates precoated with Matrigel (60 μl/well; BD Biosciences). Plates were seeded with uninduced hUCMSCs as a control. Images were captured at 3 and 6 h to record this process.

### Coculture of hUCMSCs and V-hUCMSCs for Promoting Angiogenesis *in vivo* and Examination of the Mechanism by Which hUCMSCs Promote V-hUCMSC Vasculature Formation

#### *In vivo* Matrigel Plug Assay

All of the animal experiments were approved by the Institutional Animal Care and Use Committee of Harbin Medical University (2019JS16). First, 1.0 × 10^6^ cells were resuspended in 100 μl of ice-cold Matrigel at ratios of 1:0, 1:1, and 0:1 (V-hUCMSCs/hUCMSCs). The Matrigel alone (without cells) were used as control groups. The culture mixture in Matrigel was subcutaneously injected into the right flank of each 5–7 weeks old immunodeficient mouse (CB.17 SCID, 6 mice in each group). After 1 week, the mice were sacrificed, and the Matrigel plug was removed as previously reported (Melero-Martin et al., 2008). Implants were fixed with 4% paraformaldehyde at 4°C for 24 h, embedded in paraffin, and used for hematoxylin and eosin (H&E) and immunohistochemical staining.

#### Coculture of hUCMSCs and V-hUCMSCs *in vitro* and RNA Sequencing (RNA-Seq)

hUCMSCs were seeded at 3 × 10^5^ cells per well onto transwell inserts (0.4 μm pore size; Corning, NY, United States) and incubated for 24 h to allow for initial attachment. V-hUCMSCs were seeded in six-well plates at 3 × 10^5^ cells/well into the bottom plate and incubated for 24 h to allow for initial attachment. Then, the insert with hUCMSCs was added to the six-well plates with V-hUCMSCs. The transwell coculture system was used to culture the V-hUCMSCs (VC) for 7 days. V-hUCMSCs (V) cultured in six-well plates were used as a control. The RNA-sequencing (RNA-seq) experiments were conducted in the Novogene (China). Sequencing libraries were generated using NEBNext^®^ UltraTM RNA Library Prep Kit for Illumina^®^ (NEB, United States). In brief, total RNA from V-hUCMSCs (V) or cocultured V-hUCMSCs (VC) was isolated using a TRIzol reagent following the manufacturer’s procedure, and mRNA was purified using the AMPure XP system (Beckman Coulter, Beverly, MA, United States) and reverse-transcribed to create the final cDNA library. Additionally, qRT-PCR, western blot, and immunofluorescence assays were performed with VC and V.

#### Cell Transfection

Three different HIF1A-AS2-specific siRNAs were purchased from GenePharma (Suzhou, China). The sequences are shown in Supplementary Table S3. V-hUCMSCs were cultured in six-well plates, and when the cells reached 80% confluence, the siRNAs were transfected using Lipofectamine 2000 (Invitrogen, United States), following the manufacturers’ instructions. Non-specific siRNA was used as a negative control. qRT-PCR was then performed to confirm the knockdown efficiency. After transfection for 24 h, the cells were cocultured with hUCMSCs using the transwell coculture system. The cocultured cells were harvested after 7 days and analyzed by qRT-PCR and western blot assays.

### Dental Pulp Regeneration

#### Cell Survival and Proliferation in Three-Dimensional Injectable Scaffolds *in vitro*

V-hUCMSCs and hUCMSCs were encapsulated at different concentrations (1, 2, and 3 mg/ml) in collagen I scaffolds. First, we used a live/dead cell viability kit (Molecular Probes, Inc., Eugene, OR, United States) to assess cell viability in the cell/scaffold constructs 24 h after encapsulation. The experiment was performed according to the manufacturer’s instructions, and the live and dead cells were counted (live cells were indicated by green fluorescence and dead cells by red fluorescence). Then, a cell count kit-8 (CCK-8, Dojindo, Japan) was used to evaluate cell proliferation. After cells were observed for growth on the first, third, fifth, and seventh days, the original culture medium was replaced with 100 μl of a regular medium containing 10 μl of CCK-8 solution. Five parallel replicates were prepared. After incubating cells for 4 h at 37°C, absorbance at 450 nm was determined using a spectrophotometer (Tecan).

#### Pulp Regeneration *in vivo*

The roots of freshly extracted human teeth were cut into 8–9 mm long segments. Rotary instruments (ProTaper and ProFile, Dentsply Tulsa Dental) were used to clean and shape the root canals. The root fragments were soaked in 17% EDTA for 10 min and then in 19% citric acid for 60 s. They were subsequently treated with betadine for 30 min and finally with 5.25% NaOCl for 10–15 min. Then, the fragments were rinsed with PBS and incubated in PBS at 37°C for 3–7 days (Dissanayaka et al., 2014).

The following three experimental groups were created (six root segments in each group): (1) root segments with collagen I alone; (2) root segments with collagen I + hUCMSCs; and (3) root segments with collagen I + coculture of hUCMSCs and V-UCMSCs (1:1). The cells were suspended in collagen I, and the cell/scaffold mixture was injected into the root segments, incubated overnight, and subsequently implanted into the subcutaneous space of the dorsum of 5–7 weeks old female immunodeficient mice. The implants were removed after 4 weeks, fixed in 4% formaldehyde for 48 h, and demineralized with decalcifier (Leica Biosystems) for 3 days (until the dentin offered no resistance to being cut with a blade). H&E and immunohistochemical staining was then performed to observe the structure and morphology of the regenerated pulp-like tissues.

### Statistical Analysis

All of the data are expressed as the mean ± SD of at least three separate experiments. Statistical analyses were performed using SPSS software (SPSS, United States), version 18.0. Statistical differences were determined using Student’s *t*-test or one-way ANOVA. A value of *P* < 0.05 was considered statistically significant.

## Results

### Isolation, Morphology, and Identification of hUCMSCs

#### Isolation and Morphology of hUCMSCs

**FIGURE 1:**
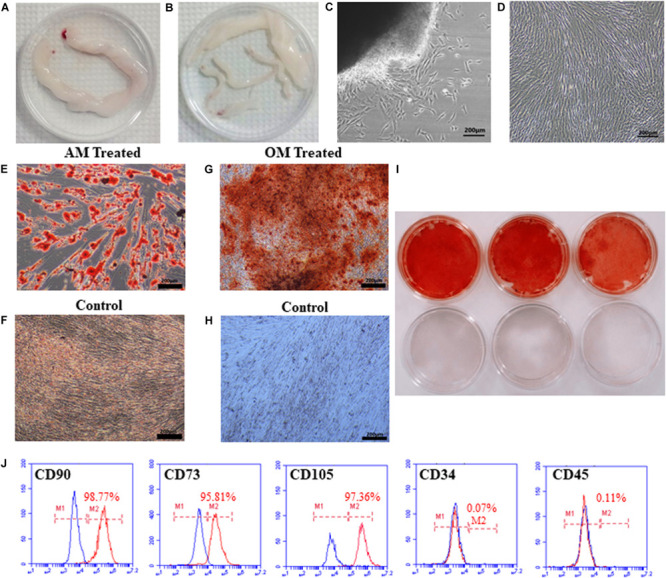
Isolation, morphology, and identification of hUCMSCs. The umbilical cord (UC) was cut into 10 cm pieces **(A)**. The blood vessels in the UC were separated to obtain WJ **(B)**. The morphology of primary cells cultured for 7 days **(C)**. The morphology of cells after passaging **(D)**. Lipid droplets were observed after culturing in adipogenic conditions for 14 days **(E)**, but not in the control groups **(F)**. Alizarin red staining showed mineralized nodules in cells grown under osteogenic conditions **(G,I)**; cells in the control group were not stained **(H,I)**. The third-generation cells were positive for CD90 (98.77%), CD73 (95.81%), and CD105 (97.36%) and negative for CD34 (0.07%) and CD45 (0.11%), as analyzed by flow cytometry **(J)**.

We isolated hUCMSCs from Wharton’s jelly of umbilical cord using tissue block culture methods (Figures 1A,B). After culturing for 7 days, the cells around the tissue blocks exhibited a fibroblast-like morphology, adherent growth, and a spindle-shaped appearance (Figure 1C). At this time, the tissue blocks that had not attached to the dish were removed. The cells gradually began to display a typical fibroblastic morphology, a dense distribution, and “whirlpool-like” growth after culturing for 15 days. Cells were passaged when they reached 80–90% confluency. After passage, the cells expanded rapidly without obvious morphological changes (Figure 1D) and were passaged once every 3–4 days thereafter.

#### Identification of hUCMSCs

After three passages, to confirm the multipotent differentiation potential of hUCMSCs, we evaluated the osteogenic and adipogenic differentiation capabilities of the cells *in vitro*. After culturing in AM for 14 days, many lipid vacuoles were observed (a unique characteristic of the adipogenic phenotype) by Oil Red O staining (Figures 1E,F). As shown in Figures 1G–I, calcium deposition, which is generally used to identify osteogenesis, was obvious in the hUCMSCs following staining with Alizarin Red S solution after induction for 14 days. The cells were then prepared for examination and analysis by flow cytometry. The cells were negative for hematopoietic lineage markers (CD34, CD45), but they expressed high levels of CD90, CD73, and CD105 (a matrix receptor) (Figure 1J). These findings demonstrated that the cells isolated in our study represented hUCMSCs and were not mixed with hematopoietic cells.

### The Influence of LE-TDM on the Biological Characteristics of hUCMSCs *in vitro*

#### *In vitro* Cytotoxicity of LE-TDM

The cytotoxicity of LE-TDM on hUCMSCs was very low, ranging from 0 to 1. According to ISO standards, LE-TDM showed good biocompatibility, indicating that it could be used as a culture medium. Most importantly, the cytotoxicity level of LE-TDM at days 1, 2, and 4 was 0, suggesting that LE-TDM could be favorable for cell proliferation. Supplementary Table S4 summarizes the relative growth rate (RGR) and cytotoxicity of LE-TDM.

#### Cell Migration Assay

**FIGURE 2:**
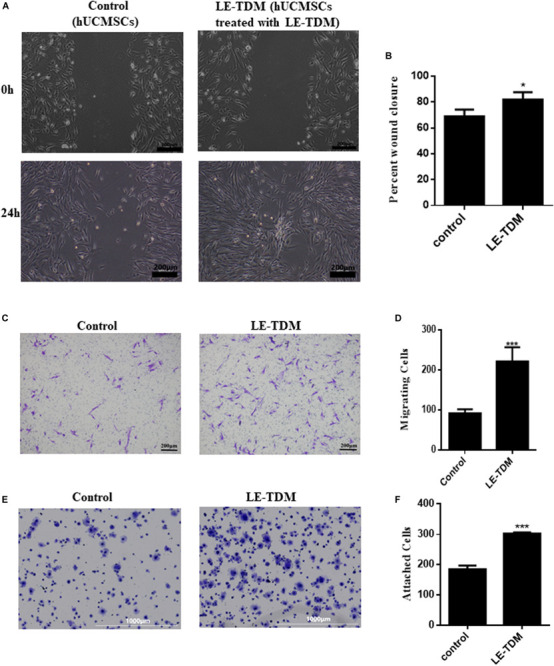
Cell motility and adhesion ability of hUCMSCs treated with LE-TDM in comparison with hUCMSCs treated with regular media. A wound healing assay was performed, and the migration of the cells into the wound area was followed by microscopy at T = 24 h **(A)**. Data are presented as the percentage of wound closure. Values are expressed as the mean ± SD (*P < 0.05) **(B)**. Migrated hUCMSCs were visualized using crystal violet **(C)**. The number of migrated cells was increased in the LE-TDM-treated group compared with that in the control group. Values are expressed as the mean ± SD (***P < 0.001) **(D)**. LE-TDM affects hUCMSC adhesion. hUCMSCs attached to Fn-coated plates were visualized using crystal violet **(E)**. The number of cells attached to Fn-coated plates was counted. Values are expressed as the mean ± SD (***P < 0.001) **(F)**.

We performed scratch and Transwell assays to assess cell motility. Interestingly, 24 h after producing a scratch in the monolayer, hUCMSCs treated with LE-TDM migrated more quickly than those cultured in a regular medium (Figures 2A,B). More hUCMSCs migrated through the basal membrane in the LE-TDM-treated group than in the control group (Figures 2C,D). Considered together, both assays showed that LE-TDM substantially induced hUCMSC migration *in vitro*.

#### Cell Adhesion Assay

The cells induced by LE-TDM for 7 days exhibited superior ability to attach to Fn-coated plates compared with cells in the control group (Figures 2E,F). The experiments confirmed that LE-TDM promoted hUCMSC attachment.

#### LE-TDM-Induced hUCMSCs Differentiated Into Odontoblast-Like Cells

**FIGURE 3:**
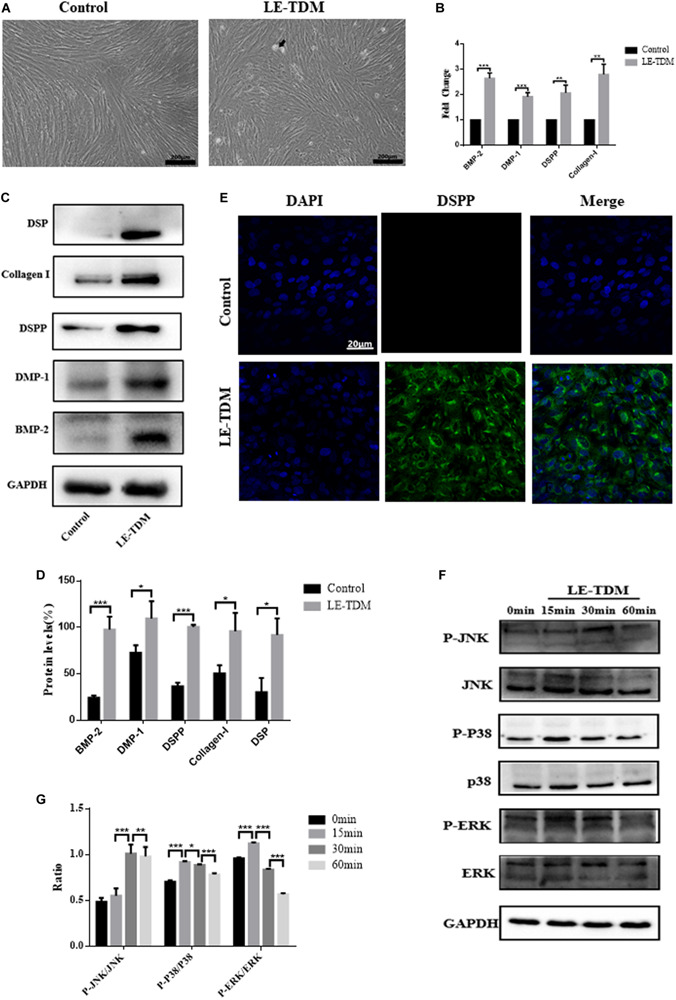
LE-TDM induces hUCMSCs to differentiate into odontoblast-like cells *in vitro*. hUCMSCs induced by LE-TDM for 7 days; the LE-TDM-induced hUCMSCs grew well, and calcified areas were observed (arrow), but their morphology was similar to that of hUCMSCs **(A)**. The effects of LE-TDM on the expression levels of DMP-1, DSPP, collagen I, and BMP-2 messenger RNA in hUCMSCs. The relative gene expression was normalized to the expression of GAPDH, and the control was set to 1.0. Values are expressed as the mean ± SD (**P < 0.01, ***P < 0.001) **(B)**. Western blot assays showed that LE-TDM-induced hUCMSCs expressed DSP, collagen I, DSPP, DMP-1, and BMP-2, whereas hUCMSCs did not express these proteins **(C)**. Values are expressed as the mean ± SD (*P < 0.05, ***P < 0.001) **(D)**. hUCMSCs cultured in LE-TDM were positively stained for DSPP, but the control cells were not **(E)**. The effects of LE-TDM on the MAPK signaling pathways for hUCMSCs. All cells were cultured for 60 min, and LE-TDM was added at 60, 30, 15, and 0 min, corresponding to the 0, 15, 30, and 60 min groups, respectively. The protein levels of ERK, p-ERK, P38, p-P38, JNK, and p-JNK were determined by western blot **(F)**. Quantitative analysis of protein bands was done by ImageJ. The levels of p-JNK/JNK, p-P38/P38, and p-ERK/ERK were normalized by GAPDH **(G)**.

hUCMSCs were cultured in LE-TDM for 7 days. The cells grew well, and their morphology did not change significantly, although calcified areas were observed (Figure 3A). qRT-PCR was used to compare the levels of BMP-2, DMP-1, DSPP, and collagen I gene expression between LE-TDM-induced hUCMSCs and non-induced hUCMSCs. BMP-2, DMP-1, DSPP, and collagen I gene expression levels were significantly upregulated in LE-TDM-induced hUCMSCs compared to controls (Figure 3B). Western blotting analysis also indicated that LE-TDM induced the expression of DSP, collagen-I, DSPP, DMP-1, and BMP-2 in hUCMSCs. These proteins showed negligible expression in non-induced hUCMSCs, indicating the differentiation of LE-TDM-induced hUCMSCs into odontoblast-like cells (Figures 3C,D). Next, we performed immunofluorescence staining for dentin sialophosphoprotein (DSPP) as a putative odontoblast-specific marker. The LE-TDM-induced hUCMSCs exhibited positive staining for DSPP, while no staining of cells in the control group was observed (Figure 3E).

#### Activation of MAPK Signaling Pathways in hUCMSCs Induced by LE-TDM

To explore whether MAPK signaling pathways are involved in the LE-TDM-mediated odontogenic differentiation process, we measured total protein and JNK, p38, and ERK protein phosphorylation by western blot. The protein levels of p-JNK gradually increased, reached a peak at 30 min, and then decreased. However, the expression levels of p-p38 and p-ERK peaked at 15 min and then subsequently decreased (Figures 3F,G).

### Endothelial Differentiation *in vitro*

**FIGURE 4:**
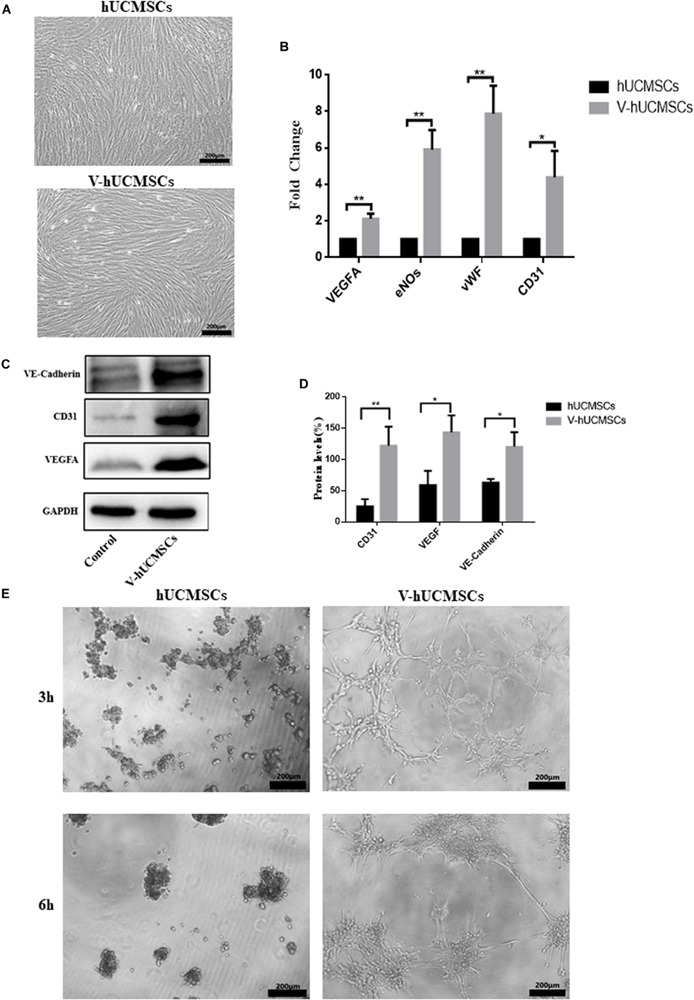
VEGF induces hUCMSC differentiation into ECs *in vitro*. hUCMSCs induced by VEGF for 7 days; the VEGF-induced hUCMSCs (V-hUCMSCs) grew well, and the morphology was similar to that of hUCMSCs, except that they exhibited a long fusiform shape with abundant cytoplasm **(A)**. The effects of VEGF on the expression levels of VEGFA, eNOs, vWF, and CD31 messenger RNA in hUCMSCs cultured for 7 days. The relative gene expression was normalized to the expression of GAPDH, and the control was set to 1.0. Values are expressed as the mean ± SD (*P < 0.05, **P < 0.01) **(B)**. Western blot assays showed that V-hUCMSCs expressed Ve-cadherin, CD31, and VEGFA, whereas the hUCMSCs did not express these proteins **(C)**. Values are expressed as the mean ± SD (*P < 0.05, **P < 0.01) **(D)**. VEGF-induced hUCMSCs formed capillary-like structures **(E)**.

After the hUCMSCs were induced by VEGF for 7 days, the cells grew well and exhibited a long fusiform shape with abundant cytoplasm without significant morphological changes (Figure 4A). Real-time PCR assays showed that V expressed the endothelial-specific genes VEGFA, eNOs, vWF, and CD31 (Figure 4B). Western blotting analysis revealed that V expressed VEGFA, CD31, and Ve-cadherin protein markers, whereas the expression levels of these markers were negligible in hUCMSCs (Figures 4C,D). To assess the ability of V to form vascular tube-like networks, non-induced hUCMSCs and V were plated on Matrigel thin films. Most of the hUCMSCs retained a round shape, while V formed vascular tube-like networks (Figure 4E).

### The Mechanism by Which hUCMSCs Promote V-hUCMSC Vascular Formation

#### *In vivo* Angiogenesis

**FIGURE 5:**
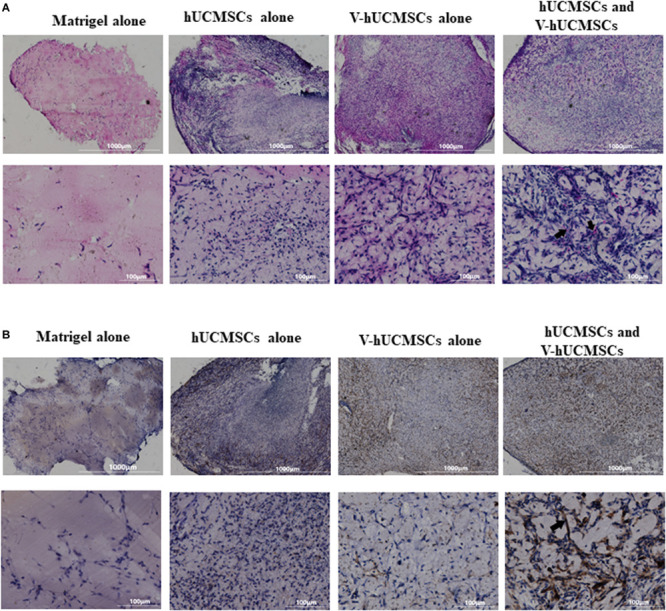
*In vivo* angiogenesis. *In vivo* Matrigel plug assay was conducted to confirm the functional role of VEGF-induced hUCMSCs (V-hUCMSCs). H&E staining revealed no vessel-like structures in the hUCMSC-only or V-hUCMSC-only group. Vessel-like structures were only observed in the hUCMSC and V-hUCMSC cotransplantation group. The presence of erythrocytes in lumen structures (arrow) suggested functional anastomoses with the host circulatory system **(A)**. Immunohistochemical staining results of CD31 showed that the vessel-like structures within the grafts stained positive for human CD31 (arrow) **(B)**.

We performed Matrigel plug assay to determine the angiogenic potential of V *in vivo*. V and hUCMSCs alone or in combination in Matrigel were injected subcutaneously into immunodeficient mice. After 7 days, there was no weight loss or behavior change in the mice. H&E staining (Figure 5A) showed that grafts with hUCMSCs alone or V alone did not contain typical vessel-like structures. However, when V and hUCMSCs were co-transplanted, robust vessel-like structures and luminal murine erythrocytes were observed in the grafts. The presence of murine erythrocytes in the lumen indicated that the newly formed vessel-like structures anastomosed with the host vasculature, resulting in perfusion within 7 days. To determine the localization of the transplanted V, immunohistochemical staining for CD31 was performed (Figure 5B). CD31-positive V was seen to be localized in an area surrounding the newly formed vessel-like structures.

#### Coculture of hUCMSCs and V-hUCMSCs *in vitro* and RNA-Seq

**FIGURE 6:**
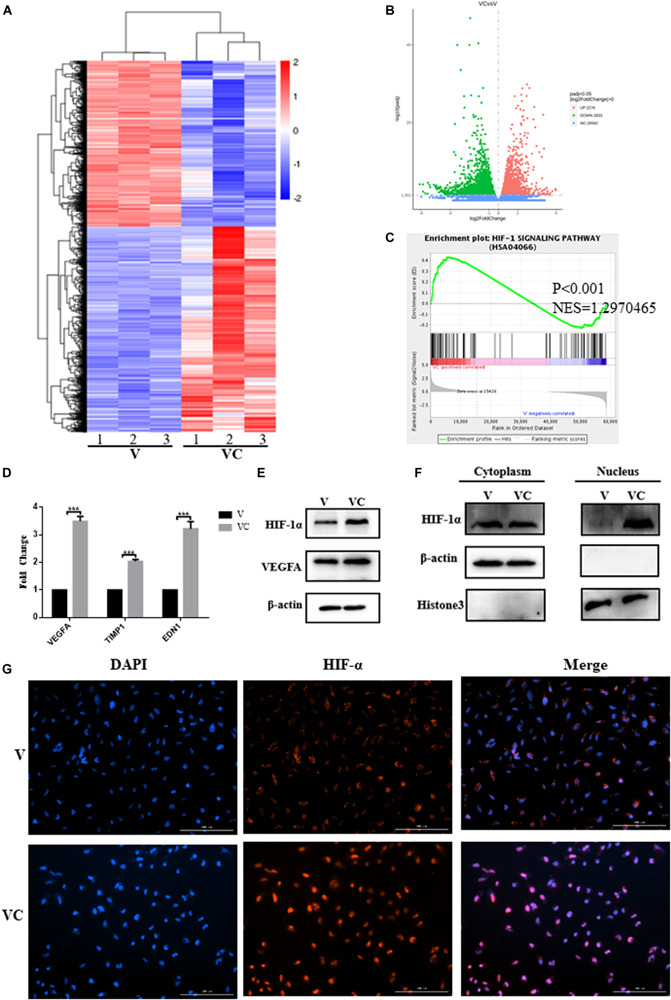
Coculture of hUCMSCs and VEGF-induced hUCMSCs (V-hUCMSCs) activated the expression of the HIF-1 signaling pathway in V-hUCMSCs. The differentially expressed genes of V-hUCMSCs (V) and cocultured V-hUCMSCs (VC) from the RNA-seq results are shown using a heatmap **(A)**. A volcano plot summarizing the RNA-seq data indicating the differentially regulated transcripts between V and VC **(B)** and a gene set enrichment analysis (GSEA) plot of the Hif-1 signaling pathway **(C)**. qRT-PCR analysis showed that coculture of hUCMSCs and V-hUCMSCs increased the mRNA (VEGFA, TIMP1, EDN1) expression in V-hUCMSCs. The relative gene expression was normalized to the expression of β-actin, and the control was set to 1.0. Values are expressed as the mean ± SD (***P < 0.001) **(D)**. Western blot was used to detect the expression of VEGFA and Hif-1α in all V and VC cells. β-actin was used as an internal control **(E)**. Western blot demonstrated that coculture significantly upregulated Hif-1α expression in the nucleus. Histone3 and β-actin were used as internal controls **(F)**. Immunofluorescence staining revealed that the expression of Hif-1α was increased in the nucleus in VC **(G)**.

To examine the mechanism by which hUCMSCs promote the angiogenesis of V, we used the Transwell coculture system to culture V-hUCMSCs and hUCMSCs. Then, whole-genome transcriptome analyses (RNA-seq) were performed for V and cocultured V-hUCMSCs (VC). The relative differential gene expression of the two groups of cells was indicated by a heat map (Figure 6A). The volcano plot revealed the overall distribution of the expression of different genes in V and VC (Figure 6B). A total of 5,109 genes showed significant differential expression between V and VC (Padj < 0.05), among which 2,276 were increased, and 2,833 were decreased in VC. To determine the biological pathways that might be involved in hUCMSC-induced promotion of V vascular formation, we performed gene set enrichment analysis (GSEA). The results indicated that VC exhibited high hypoxia-inducible factor 1 (Hif-1) signaling pathway activity, suggesting that hUCMSCs could activate the Hif-1 signaling pathway in VC (Figure 6C).

#### Identification of Differentially Expressed Genes

**FIGURE 7:**
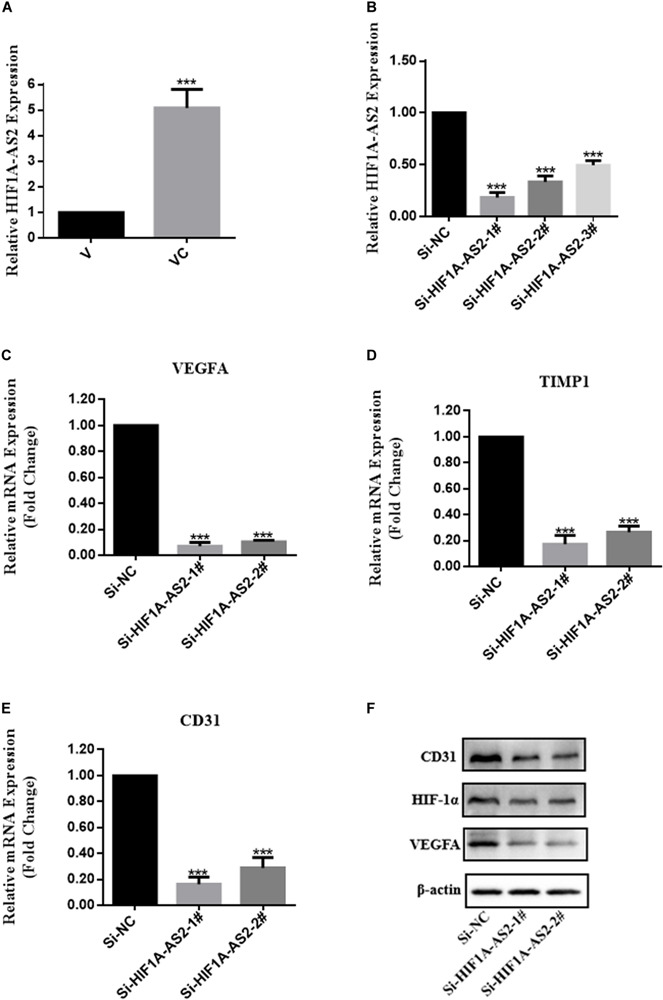
Expression profiles of HIF1A-AS2 in VC (cocultured V-hUCMSCs) and the association between HIF1A-AS2 and the HIF-1 signaling pathway in VC. HIF1A-AS2 expression in V and VC was analyzed by qRT-PCR. The levels of HIF1A-AS2 in VC were normalized to that in V **(A)**. Relative HIF1A-AS2 expression in VC transfected with HIF1A-AS2 siRNAs **(B)**. Relative mRNA expression of genes involved in the Hif-1 signaling pathway in HIF1A-AS2-deficient VC was analyzed by qRT-PCR. **(C–E)** The relative level of gene expression was normalized against β-actin, and the control was set to 1.0. Values are expressed as the mean ± SD (***P < 0.001). VEGFA, Hif-1α, and CD31 protein expression in HIF1A-AS2-deficient VC was assessed by western blot **(F)**.

To validate the RNA-seq results, qRT-PCR was performed to compare the fold changes in expression according to RNA-seq with those according to qRT-PCR. The qRT-PCR results showed that the expression of VEGFA, TIMP1, and EDN1 differed significantly between VC and V, and the trend was consistent with the sequencing results, thus indicating that the RNA-seq results were highly reliable (Figure 6D). In addition, western blotting analysis demonstrated that VEGFA and Hif-1α expression in VC was upregulated in comparison to the levels in V (Figure 6E). Considered together, these results indicated that hUCMSCs activated the Hif-1 signaling pathway in VC. To explore the mechanism underlying hUCMSC-mediated Hif-1 signaling in VC, we determined the subcellular localization of Hif-1α in VC. Immunofluorescence analysis showed that hUCMSCs increased the translocation of Hif-1α to the nucleus, compared to the control group (Figure 6G). Western blotting analysis showed similar hUCMSC-mediated changes in the expression of Hif-1α in the cytoplasm and nucleus (Figure 6F). Surprisingly, significantly higher expression of long non-coding RNA (LncRNA) HIF1A-AS2 was seen in VC than in V, according to the qRT-PCR data (Figure 7A).

#### hUCMSCs Promote Angiogenesis of V-hUCMSCs Through the Long Non-coding RNA HIF1A-AS2 Activation of the Hif-1 Signaling Pathway

To further explore the influence of HIF1A-AS2 on the activation of the Hif-1 signaling pathway in VC, we constructed a small interfering RNA (siRNA) and performed transient transfection to knock down HIF1A-AS2 in VC (VC-siRNA). The gene has three different targets in the CDS zone. As shown in Figure 7B, HIF1A-AS2 expression was more efficiently silenced in VC by si-HIF1A-AS2-1# and si-HIF1A-AS2-2# according to the qRT-PCR data. Therefore, we used si-HIF1A-AS2-1# and si-HIF1A-AS2-2# to investigate the potential mechanisms in subsequent experiments. To further investigate the function of HIF1A-AS2 in the activation of the Hif-1 signaling pathway in VC, we performed qRT-PCR and western blot assays. The qRT-PCR results showed that the expression levels of VEGFA, TIMP1, and CD31 were reduced after HIF1A-AS2 knockdown by siRNAs (Figures 7C–E). In addition, Western blotting analysis demonstrated that knockdown of HIF1A-AS2 reduced VEGFA, Hif-1α, and CD31 expression in VC (Figure 7F).

### Pulp Regeneration *in vivo*

#### Cell Survival and Proliferation in Collagen I Scaffolds

**FIGURE 8:**
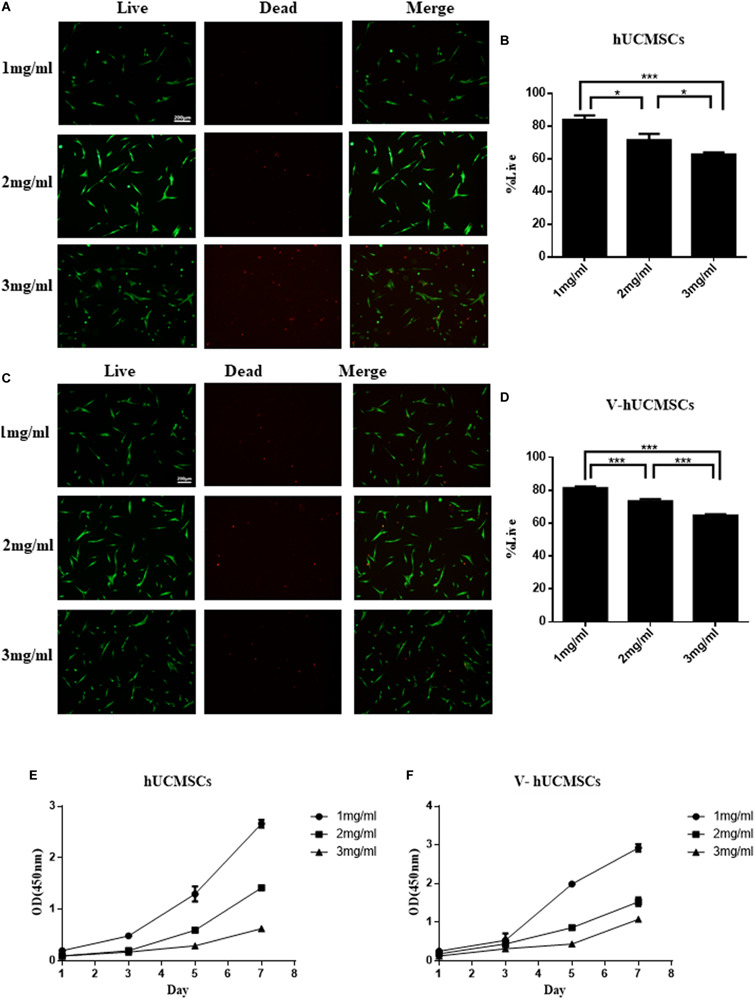
Cell survival and proliferation in three-dimensional injectable scaffolds *in vitro*. Live/dead assays were performed after the cells were encapsulated in 1, 2, and 3 mg/ml collagen I for 24 h, and the cells were counted by cell image analysis software (ImageJ). The cells in the 1 mg/ml group showed a higher rate of cell survival than those in the 2 and 3 mg/ml groups. Values are expressed as the mean ± SD (*P < 0.05, ***P < 0.001) **(A–D)**. CCK-8 assays were performed to quantitatively evaluate cell viability from days 1 to 7. Cell viability in the 1 mg/ml group was higher than in the 2 and 3 mg/ml groups **(E,F)**.

The live/dead assay indicated that 1 mg/ml collagen I was the optimal concentration for hUCMSCs and V, compared to 2 and 3 mg/ml collagen I (Figures 8A–D). The CCK-8 assay showed that hUCMSCs and V proliferated faster in the 1 mg/ml group than in the 2 and 3 mg/ml groups (Figures 8E,F). Therefore, collagen I was used at a concentration of 1 mg/ml in subsequent experiments.

#### Pulp-Like Tissue Regeneration *in vivo*

**FIGURE 9:**
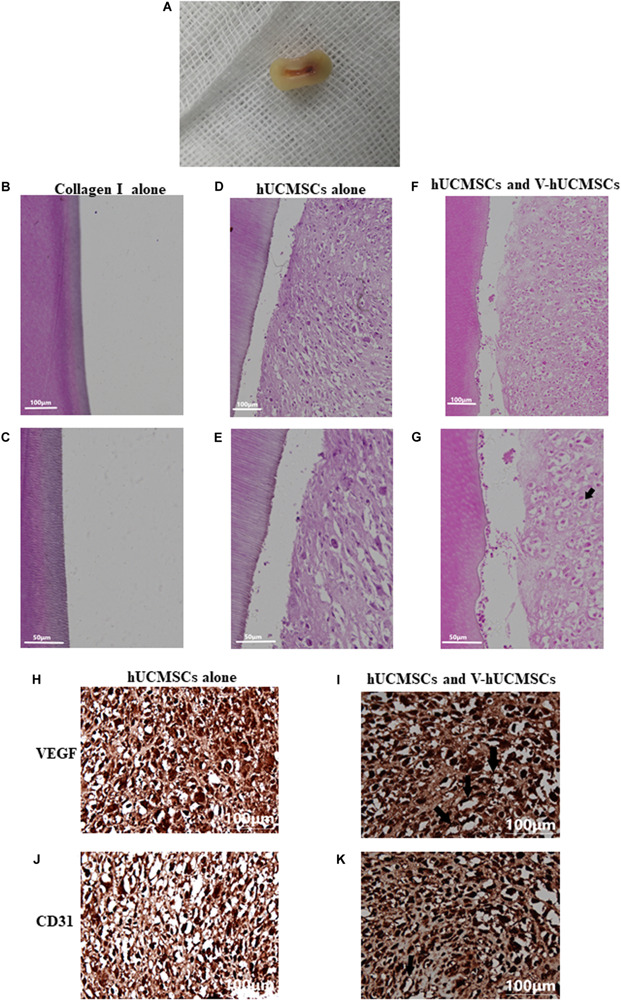
Pulp regeneration *in vivo*. Macroscopic view of the root segment **(A)**. Root segments with collagen I alone did not contain any tissue **(B,C)**. hUCMSCs alone in collagen I revealed loosely arranged connective tissue **(D,E)**. Significantly increased extracellular matrix deposition was observed in the hUCMSC and VEGF-induced hUCMSC (V-hUCMSCs) groups **(F,G)**. The hUCMSC and V-hUCMSC cocultured groups exhibited an angiogenesis tendency (**G,I,K**, arrows). There was no angiogenesis in hUCMSCs alone group **(H,J)**.

The collagen I group did not exhibit the regeneration of any pulp-like tissue (Figures 9B,C) for 4 weeks after transplantation. Interestingly, in this group, no obvious mouse subcutaneous tissue was detected growing in the root canals. These results suggested that collagen I does not have the ability to attract endogenous cells to populate the scaffold. However, all of the cell transplantation groups (monoculture and coculture groups) showed regeneration of pulp-like tissue in the root canals (Figures 9A,D–G). Importantly, the coculture group showed more extensive extracellular matrix formation with large amounts of collagen deposition compared to the monoculture group, which exhibited loosely arranged tissue with less cellularity and matrix material. In addition, the cocultured group exhibited some degree of angiogenesis (Figures 9I,K, arrows). However, none of the groups showed new dentin formation along the dentinal wall of the root canal.

## Discussion

Previous investigations have indicated that human umbilical cord tissue can be acquired non-invasively, and it is associated with a low risk of infection, so it can be considered an attractive seed cell source for tissue engineering (Han et al., 2011; Peng et al., 2011). In this study, we isolated many adherent cells from human umbilical cord Wharton’s jelly that exhibited a typical fibroblast-like morphology. Flow cytometry analysis showed that umbilical cord–derived cells expressed specific surface markers of mesenchymal cells rather than hematopoietic stem cell markers, consistent with previous studies (Chen et al., 2009). Our results also showed that umbilical cord–derived cells maintained the potential to differentiate into adipocytes and osteoblasts when induced by the appropriate conditioned medium. Considering these factors, the cells isolated from human umbilical cord Wharton’s jelly in the present study were identified as hUCMSCs (Han et al., 2011).

Studies have shown that different types of stem cells require a specific biomimicking microenvironment to drive their differentiation toward a specific cell lineage (Yang et al., 2009; Fu and Ohnishi, 2018). Recent studies have shown that tooth development follows a strict process, and many types of growth factor and signaling molecules are involved in the process of tooth development (Huo et al., 2010; Inanc and Elcin, 2011). It has been reported that the organic matrix of dentin contains large amounts of growth factors, non-collagenous protein, and collagen protein, and many of these factors and proteins have been confirmed to be essential for dentin development, mineralization, and regeneration (Marshall et al., 1997; Park et al., 2009). In addition, previous studies have indicated that a treated dentin matrix secretes a mixture of soluble factors, thus creating an odontogenic microenvironment that induces stem cell differentiation into odontoblasts (Huang et al., 2006; Wu et al., 2008; Liu et al., 2016). Therefore, based on the above studies, we believe that LE-TDM can be used to induce hUCMSCs to differentiate into odontoblast-like cells.

In this study, cytotoxicity tests first indicated that LE-TDM has good biocompatibility for hUCMSCs. The cell mobility test indicated that hUCMSCs have enhanced migration ability when exposed to LE-TDM. Previous studies have reported that cell attachment, adhesion, and spreading are the first steps in cell material interactions, affecting cell differentiation (Cronin et al., 2004; Yamaguchi et al., 2004). In this study, the LE-TDM group showed better cell adhesion ability, suggesting that LE-TDM plays an important role in promoting hUCMSC differentiation.

This study also demonstrated that LE-TDM-induced hUCMSCs formed calcified nodules, indicating that, under the microenvironment induced by LE-TDM, hUCMSCs can differentiate into the odontogenic/osteogenic lineage. Furthermore, the crucial odontogenic phenotype was evaluated by DSPP immunostaining and the expression of DSP, collagen I, DSPP, DMP-1, and BMP-2 proteins. In addition, this gene expression was detected at the mRNA level. Consistent with our hypothesis, the evidence presented above suggested that LE-TDM maintains odontogenic potential, and that LE-TDM can confer the capacity of hUCMSCs to differentiate toward the odontogenic lineage *in vitro*. Therefore, LE-TDM appears to constitute a suitable *in vitro* conditioning medium for hUCMSCs. With the induction of proteins and factors secreted by the dentin matrix, hUCMSCs that are subsequently transplanted into root canals can adhere to the dentinal surface and differentiate into odontoblasts. Therefore, hUCMSCs may be used as seed cells in pulp regeneration, pulp capping, and tooth regeneration in future studies.

This study showed that hUCMSCs possess the ability to differentiate into endothelial-like cells, both *in vitro* and after transplantation *in vivo*. V showed the biological characteristics of ECs, including expression of endothelial markers and network formation. However, transplantation of hUCMSCs or V alone did not result in the formation of vessel-like structures *in vivo*. Rather, when hUCMSCs and V were cotransplanted, many vessel-like structures were observed, along with host erythrocytes in the lumen. We speculated that the interactions between hUCMSCs and V might be involved in the process of angiogenesis. These results suggested that hUCMSCs could serve as a promising cell source for vascular tissue regeneration, treatment of endothelial dysfunction, or dental pulp regeneration.

To examine the mechanism by which hUCMSCs promote the angiogenesis of V, we used a transwell coculture system to culture V and hUCMSCs. Then, whole-genome transcriptome analysis (RNA-seq) was performed on V and VC. GSEA showed that VC exhibited high Hif-1 signaling pathway activity. And the qRT-PCR results were consistent with the sequencing results. Considered together, these results demonstrated that hUCMSCs activated the Hif-1 signaling pathway in VC. Surprisingly, qRT-PCR analysis showed that the expression level of the LncRNA RNA HIF1A-AS2 was significantly higher in VC than in V. LncRNAs are non-coding RNAs; they play roles in histone modification, direct transcriptional regulation, and blocking of the binding of transcription factors to promoters (Khalil et al., 2009; Guttman and Rinn, 2012). Our study further revealed that HIF1A-AS2 is associated with angiogenesis of VC. HIF1A-AS2 knockdown inhibited the expression of VEFGA and HIF-1α in VC; the expression of CD31 was also inhibited. Therefore, we reveal a new underlying mechanism of hUCMSCs to promote the angiogenesis of V-hUCMSCs. When V was cocultured with hUCMSCs, hUCMSCs facilitated the upregulation of HIF1A-AS2 expression in V, thus activating the Hif-1 signaling pathway and ultimately promoting the angiogenesis of V.

Finally, following dental pulp regeneration *in vivo*, both of the cell transplantation groups (monoculture and coculture groups) exhibited pulp-like tissue regeneration in the root canals. The coculture groups displayed enhanced extracellular matrix formation with larger amounts of collagen deposition than the monoculture group, but there was only small amount of angiogenesis without luminal murine erythrocytes. In the pulp regeneration experiment, we used collagen I as a scaffold material. However, in the *in vivo* angiogenesis experiment, vessel-like structures formed and luminal murine erythrocytes were observed in the grafts when using Matrigel. The scaffold is an important element in tissue engineering. An appropriate scaffold for dental pulp regeneration should at least be biocompatible and promote the regeneration of multiple pulp tissues. Although collagen I has good biocompatibility for V and hUCMSCs, it lacks the property of inducing angiogenesis. These findings suggest the importance of using appropriate scaffolds for dental pulp tissue engineering. Moreover, neither group showed the odontoblasts and new dentin formation along the dentinal wall of the root canal, possibly due to the shrinkage of the collagen I scaffold leading to the absence of contact between the cells and the dentinal wall. Without the induction of the dentin wall, the hUCMSCs did not differentiate into odontoblasts. Previous studies have shown that Paino et al. (2017) fabricated woven bone samples by DPSCs, then transplanted samples into the rats, remodeled into vascularized bone tissue, and did not need the use of a scaffold. Hanna et al., controlled calcium oscillations in mesenchymal stem cells using microsecond pulsed electric fields. These studies encouraged us to find a new method to regulate the differentiation of hUCMSCs, such as the control of calcium oscillations or fabricated woven bone samples without the use of a scaffold (Hanna et al., 2017).

## Conclusion

**FIGURE 10:**
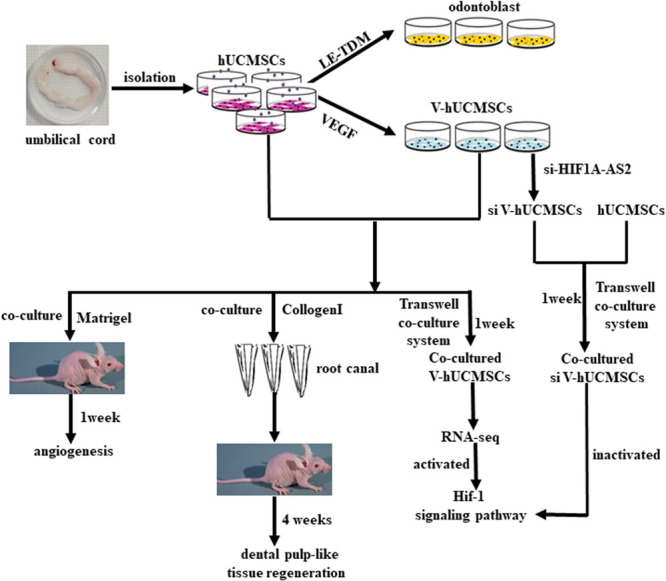
Schematic model of the experimental process.

In summary, the results of the present study showed that hUCMSCs possess the ability to differentiate into odontoblast-like cells under the microenvironment induced by LE-TDM *in vitro*. V was shown to be functional ECs. Matrigel plug assay showed that cocultured hUCMSCs and V formed vessel-like structures. GSEA showed that VC exhibited high Hif-1 signaling pathway activity, suggesting that the Hif-1 signaling pathway might be activated in VC. Cotransplantation of hUCMSCs and V can regenerate dental pulp-like tissue *in vivo* (Figure 10). Therefore, hUCMSCs might be used as a cell source for rapid clinical translation in the field of vascular regeneration, for the treatment of endothelial dysfunction, and for dental pulp tissue engineering. Further studies are required to identify a more suitable scaffold for hUCMSCs to allow for effective regeneration of the pulp dentin complex.

## Data Availability Statement

The datasets generated for this study can be found in the NCBI SRA BioProject ID is: PRJNA631150.

## Ethics Statement

The animal study was reviewed and approved by the Institutional Animal Care and Use Committee of the Harbin Medical University.

## Author Contributions

SZ, SP, and YN conceived, designed, coordinated, and directed the experiments. SZ and SP wrote the manuscript. SZ, HD, and XY carried out the cell experiments and performed the statistical analysis. WZ, YL, LR, and XG revised the manuscript. All authors reviewed and approved the final manuscript.

## Conflict of Interest

The authors declare that the research was conducted in the absence of any commercial or financial relationships that could be construed as a potential conflict of interest.
