# Supplementary material for: Human Umbilical Cord Mesenchymal Stem Cell Differentiation Into Odontoblast-Like Cells and Endothelial Cells: A Potential Cell Source for Dental Pulp Tissue Engineering
Source: Front Physiol. 2020 Jun 23;11:593. doi: 10.3389/fphys.2020.00593 (PMC7344301; doi:10.3389/fphys.2020.00593)
Supplement: Supplementary file 1 [file Table_1.DOCX]

**Table. S1** Cytotoxicity levels scored accordingto the ISO standard.

| RGR(%) Cytotoxicity |
| --- |
| ≥100 0  75-99 1 |
| 50-74 2 |
| 25-49 3 |
| 1-24 4 |
| 0 5 |

**Table. S2** Sequences of primers used for real-time PCR analysis

| Gene | Primer | Sequence( 5′–3′) |
| --- | --- | --- |
| GAPDH  BMP-2 | Forward  Reverse  Forward  Reverse | GAAGGCTGGGGCTCATTT  CAGGAGGCATTGCTGATGAT  GACGTTGGTCAACTCTGTTAAC  GTCAAGGTACAGCATCGAGATA |
| DSPP | Forward Reverse | CCCGTAAGATTCCTTTTGTGA  TTCCCCCAGTTGTTTTTGTT |
| DMP-1 | Forward Reverse | GTGAGTGAGTCCAGGGGAGATAA  TTTTGAGTGGGAGAGTGTGTGC |
| Collagen I | Forward Reverse | TCCGACCTCTCTCCTCTGAA  TGCTTTGTGCTTTGGGAAGT |
| VEGFA | Forward Reverse | ATCGAGTACATCTTCAAGCCAT GTGAGGTTTGATCCGCATAATC |
| CD31 | Forward Reverse | CCTTGGAAATTGGAAGAGCA  CTGTGTATGAGGGTGCATGG |
| eNOs    vWF  β-actin    EDN1  TIMP1  HIF1A-AS2 | Forward Reverse  Forward Reverse Forward  Reverse Forward  Reverse Forward  Reverse  Forward Reverse | ATGCTCCCAACTTGACCATC  TGTCCTGCACGTAGGTCTTG  GAAGCAGACGATGGTGGATT  CACAGGAGCAGGTGTCGTAA  TGGCACCCAGCACAATGAA  CTAAGTCATAGTCCGCCTAGAAGCA  TAGCCAAAAAGACAAGAAGTGC  TTCTTCCTCTCACTAACTGCTG  CATCACTACCTGCAGTTTTGTG  TGGATAAACAGGGAAACACTGT  TGAGTTGGAGGTGTTGAAGCA  ACATCTTCTGTGGACCAGGC |

**Table. S3** Sequences of the siRNAs used in this study.

| Gene name /  siRNA | | Sequences (5’→3’) |
| --- | --- | --- |
| HIF1A-AS2  siRNA1-sense  siRNA1-antisense  siRNA2-sense  siRNA2-antisense  siRNA3-sense  siRNA3-antisense  NC | CAGGAAACUUAAGCUUACATT  UGUAAGCUUAAGUUUCCUGTT  GGGAUAUUAUGGUUGUUAUTT  AUAACAACCAUAAUAUCCCTT  CUCAGGGUAAAGGACCUAATT  UUAGGUCCUUUACCCUGAGTT | |
| siRNA-sense | UUCUCCGAACGUGUCACGUTT | |
| siRNA-antisense | ACGUGACACGUUCGGAGAATT | |

**Table. S4** Cytotoxicity levels and RGR of LE-TDM

| Time(Days) | RGR(%) | Cytotoxicity |
| --- | --- | --- |
| Day1 | 103.54 | 0 |
| Day2 | 116.89 | 0 |
| Day3 | 90.20 | 1 |
| Day4 | 81.65 | 0 |
| Day5 | 94.71 | 1 |
